# Surgical margin status and its impact on prostate cancer prognosis after radical prostatectomy: a meta-analysis

**DOI:** 10.1007/s00345-018-2333-4

**Published:** 2018-05-15

**Authors:** Lijin Zhang, Bin Wu, Zhenlei Zha, Hu Zhao, Jun Yuan, Yuefang Jiang, Wei Yang

**Affiliations:** Department of Urology, Affiliated Jiang-yin Hospital of the Southeast University Medical College, 163 Shou-shan Road, Jiangyin, 214400 Jiangsu People’s Republic of China

**Keywords:** Prostate cancer, Radical prostatectomy, Positive surgical margin, Prognosis, Meta-analysis

## Abstract

**Background and purpose:**

Positive surgical margins (PSMs) correlate with adverse outcomes in numerous solid tumours. However, the prognostic value of PSMs in prostate cancer (PCa) patients who underwent radical prostatectomy remains unclear. Herein, we performed a meta-analysis to evaluate the association between PSMs and the prognostic value for biochemical recurrence-free survival (BRFS), cancer-specific survival (CSS), overall survival (OS), cancer-specific mortality (CSM) and overall mortality (OM) in PCa patients.

**Materials and methods:**

According to the PRISMA statement, online databases PubMed, EMBASE and Web of Science were searched to identify relevant studies published prior to February 2018. The hazard ratios (HRs) and 95% confidence intervals (95% CIs) were calculated to evaluate the relationship between PSMs and PCa.

**Results:**

Ultimately, 32 cohort studies that met the eligibility criteria and involved 141,222 patients (51–65,633 per study) were included in this meta-analysis. The results showed that PSMs were significantly predictive of poorer BRFS (HR = 1.35, 95% CI 1.28–1.48, *p* < 0.001), CSS (HR = 1.49, 95% CI 1.16–1.90, *p* = 0.001) and OS (HR = 1.11, 95% CI 1.02–1.20, *p* = 0.014). In addition, PSMs were significantly associated with higher risk of CSM (HR = 1.23, 95% CI 1.16–1.30, *p* < 0.001) and OM (HR = 1.09, 95% CI 1.02–1.16, *p* = 0.009) in patients with PCa.

**Conclusions:**

Our study suggests that the presence of a histopathologic PSM is associated with the clinical outcomes BRFS, CSS, OS, CSM and OM in patients with PCa, and PSMs could serve as a poor prognostic factor for patients with PCa.

**Electronic supplementary material:**

The online version of this article (10.1007/s00345-018-2333-4) contains supplementary material, which is available to authorized users.

## Introduction

In 2016, prostate cancer (PCa) was the most common newly diagnosed cancer in males, with 1.6 million new cases per year, and 26,730 men died from PCa, which was the third leading cause of cancer death in males [1]. With the wide use of prostate-specific antigen (PSA) screening and increased public awareness of PCa, 90% of patients are being diagnosed with localised PCa [2]. Despite effective treatments with curative intent such as radical prostatectomy (RP), up to 30% of patients will experience biochemical recurrence (BCR), of which 20‒30% will progress to clinical metastasis or death [3]. To date, there have been a number of studies performed to identify histological parameters associated with prognostic outcomes after RP, which might lead to more informative prognostic information in patient monitoring.

A positive surgical margin (PSM) is determined by the stained areas of soft tissue on the RP specimen. The incidence of PSMs is influenced by the presence of extra-prostatic extension, with a rate that ranges from 10 to 48% [4]. Despite improvements in surgical techniques and standardisation of the RP procedure, PSMs remain an active area of investigation regarding the variability among surgeons and institutions. Several studies have shown that PSMs can predict metastatic progression [5] and/or local recurrence and distant metastasis [6, 7], whereas other studies have shown no such relationship [8, 9].

Therefore, to further clarify the prognostic value of PSMs in PCa, we performed this meta-analysis based on all published epidemiological studies to evaluate whether the presence of a PSM has a prognostic impact on biochemical recurrence-free survival (BRFS), cancer-specific survival (CSS), overall survival (OS), cancer-specific mortality (CSM) and overall mortality (OM) in patients with PCa.

## Materials and methods

### Literature search

According to the guidelines of the preferred reporting items for systematic reviews and meta-analyses (PRISMA) [10], we searched PubMed, EMBASE and Web of Science from their inception to February 2018. Because the studies included in this meta-analysis have been published, no ethical approval was required. MeSH terms and free words searched for were as follows: ‘prostate cancer OR prostate neoplasm’, ‘radical prostatectomy’, ‘positive surgical margin’, ‘survival outcome’, ‘prognosis’ and their combinations. The reference lists of previous relevant reviews were also manually checked to identify all available studies. The language of the publications was restricted to English.

### Inclusion and exclusion criteria

The eligible studies were included only if they met the following criteria: (1) clinical trials that reported patients with PCa; (2) PSM status that was assessed by pathologists; (3) survival outcomes (BRFS, CSS, OS, CSM and OM) of patients with PSMs that were compared with those of patients with negative surgical margins; (4) results that were reported as risk estimate hazard ratios (HRs) with corresponding 95% confidence intervals (95% CIs), or sufficient data that was provided to estimate these measures; and (5) the adoption of only the more well-designed, recent and informative publication in this meta-analysis when more than one study analysed the same patient cohort. Accordingly, studies with the following criteria were excluded: (1) reviews, meeting abstracts, letters, case reports, author replies and articles not on humans; (2) studies not related to PCa; (3) studies that did not analyse the presence of a PSM and the clinical features and survival outcomes; and (4) studies lacking sufficient data to acquire HRs and 95% CIs.

### Data extraction and quality assessment

The following data of the eligible studies were extracted independently by two reviewers (ZLZ and HZ): first author, publication year, country, sample size, recruitment period, age of patients, preoperative PSA, histopathological subtype, follow-up time, and survival end point. All discrepancies in data extraction were resolved by discussion between the two reviewers or consultation with a third reviewer (BW). The quality of the included studies was assessed using the Newcastle–Ottawa scale (NOS) [11] for nonrandomized studies. Each study was assessed by eight methodological items with a score ranging from 0 to 9. Studies with scores of six or higher were graded as high quality. Only high-quality studies were included for further analysis to assure the quality of this meta-analysis.

### Statistical analysis

Pooled HRs with 95% CIs were used to evaluate the association of a PSM with PCa prognosis and clinicopathological characteristics. An observed HR > 1 indicated a poor prognosis for patients with PSMs. Heterogeneity between studies was assessed using the *Q* and *I*^2^ statistics. *p *< 0.10 or *I*^2^ > 50% were used to indicate heterogeneity. A random-effect (RE) model was used when heterogeneity was observed (*p* < 0.1); otherwise, a fixed-effect (FE) model was used. To obtain a more precise evaluation of heterogeneity, subgroup analysis was performed for BRFS, CSS, OM and OS based on geographical region, date of publication, mean age, sample size, mean preoperative PSA (p-PSA) concentration, median follow-up and adjuvant radiotherapy (aRT). Sensitivity analysis was performed to test the reliability of the total pooled results by sequential omission of individual studies. In addition, publication bias was assessed using funnel plots and Egger’s test. All statistical tests in this meta-analysis were undertaken using Stata 14.0 software (Stata Corporation, College Station, TX). All statistical tests were two-tailed, and *p *< 0.05 was considered statistically significant.

## Results

### Search results

**Fig. 1:**
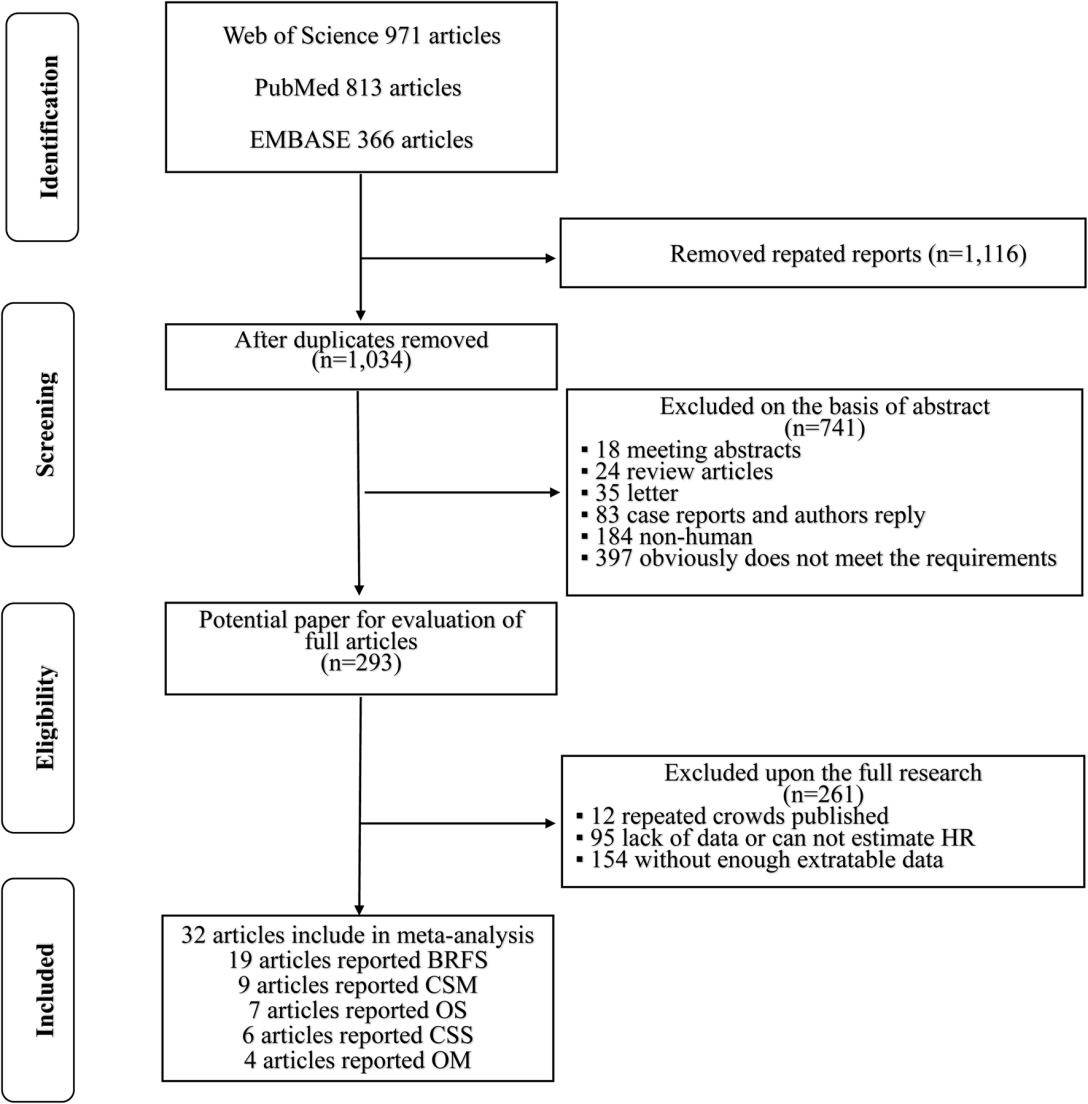
Flow chart of study selection in this meta-analysis

Figure 1 shows a flow chart of our selection process. The search strategy yielded 2150 potential studies. According to the exclusion criteria, we excluded 1857 duplicate or not relevant articles on screening of the titles and abstracts. The full text of 293 articles was assessed, and 256 articles were excluded for study groups or insufficient data. Finally, 32 publications [8, 12–42] (19 reporting BRFS, 9 CSM, 7 OS, 6 CSS, 4 OM) published from 2010 to 2017 were included in the meta-analysis.

### Study characteristics and quality assessments

The detailed characteristics of the studies are listed in Table 1. All studies were published between 2010 and 2017, with the mean duration of follow-up varying from 18.1 to 174 months. A total of 141,222 patients (ranging from 51 to 65,633) underwent RP for PCa management, of which 31,421 patients were reported to have PSMs. Nine studies [8, 17, 19, 20, 30, 32, 34, 36, 37, 42] reported the use of radiotherapy as an adjuvant therapy after RP, and the proportion of patients who received aRT was 0.2–69%. Of the 32 studies, 11 were conducted in North America, 10 in Asia, 8 in Europe and 3 at international multi-centres. All articles included were published in English. The NOS was applied to assess the quality of the included studies, and the results showed all the studies were of high quality, with an NOS score ≥ 7 (Supplementary Table S1).

**Table 1:** Main characteristics of the eligible studies

Author	Year	Country	No. of patients	Recruitment period	Age (years)	p-PSA (ng/ml)	Specimen GS ≦ 7/> 7	Pathological stage1–2/3–4	SM+/SM−	Follow-up (months)	Survival analysis
Kliment et al. [12]	2017	Slovak Republic	114	1995–2012	Mean ± SD 62.6 ± 5.9	Median (range) 10.5 (3.2–100)	58/56	0/114	64/50	Median (range) 62 (4–205)	BRFS
Fujimura et al. [13]	2017	Japan	908	2005–2016	Median (range) 67 (47–80)	Median 7.9	777/130	650/258	302/606	NA	BRFS
Heering et al. [14]	2017	Denmark	6857	1995–2011	Median (IQR) 64.1 (60.3–67.6)	Median (IQR) 8.9 (6.2–13.0)	6127/592	4812/2565	1481/4805	Median 76.8	CSM
Zhang et al. [15]	2016	China	205	2009–2013	Median (IQR) 68 (62–73)	Median (IQR) 13.1 (7.9–17.7)	171/34	118/87	51/154	Median (range) 43.8 (2–60)	BRFS
Xu et al. [16]	2016	China	243	2005–2010	Mean ± SD 68 ± 7.04	Mean ± SD 13.99 ± 10.21	204/39	219/24	37/206	Median (range) 61 (7–97)	BRFS
Moschini et al. [17]	2016	USA	1011	1987–2012	NA	Median 12.0	647/364	355/657	566/445	Median 211.2	CSM, OM
Raheem et al. [18]	2016	Korea	800	2005–2010	Mean ± SD 64.3 ± 7.4	Median (IQR) 7.2 (5–12)	628/172	487/313	293/507	Median (IQR) 57 (23.2–65.8)	CSS
Moris et al. [19]	2016	USA	1249	1989–2011	Median (IQR) 66 (60–70)	Median (IQR) 18.2 (8.1–33)	807/442	300/949	671/578	Median (IQR) 24.3 (11–56)	CSS, OS
Boehm et al. [20]	2016	European	8741	1992–2009	NA	Median (IQR) 6.5 (4.7–9.7)	8465/276	6187/2553	1541/7200	Median (IQR) 65.6 (48.3–96.7)	CSM
Mithal et al. [8]	2016	Multi-centres	4051	1988–2013	Mean 62	NA	3561/490	3069/982	1600/2451	Median (IQR) 79.2 (38.4–127.2)	CSM, OS
Maxeiner et al. [21]	2016	Germany	441	1999–2007	Median (IQR) 63 (59–66)	Median (IQR) 8.3 (5.3–13)	293/148	422/19	113/328	Median (IQR) 81.9 (59.7–108.7)	BRFS
Eminaga et al. [22]	2016	Multi-centres	1180	NA	Median (range) 61 (35–80)	Mean ± SD 8.63 ± 8.36	1065/107	NA	347/666	Median 60	BRFS, CSS, OS
Liu et al. [23]	2015	Japan	160	2007–2010	NA	NA	125/35	94/66	15/155	Median (range) 51 (6–76)	BRFS
Kim et al. [24]	2015	Korea	613	2005–2013	Median (range) 66 (44–89)	Median (range) 8 (1–79)	202/79	544/79	151/462	Median (range) 44 (12–154)	BRFS
Jeong et al. [25]	2015	USA	15,565	1982–2012	Mean ± SD 58.3 ± 7.8	Mean ± SD 6.8 ± 5.8	13,277/2288	NA	2132/13,433	Median (range) 108 (12–324)	BRFS, CSS, OS
Rouanne et al. [26]	2014	France	403	1988–2001	Median (range) 66 (46–81)	Median (range) 10 (0.5–158)	340/63	403/0	108/295	Median (range) 147 (126–251)	BRFS
Park et al. [27]	2014	Korea	1007	2007–2012	NA	NA	838/169	634/373	228/779	Median (IQR) 32 (15.6–45.9)	BRFS
Knoedler et al. [28]	2014	USA	18,916	1987–2009	Median (IQR) 63 (58–68)	Median (IQR) 6.3 (4.4–9.8)	12,469/993	10,258/3425	4007/14,909	Median (IQR) 112.8 (60–174)	CSM, OM
Touijer et al. [29]	2014	USA	369	1988–2010	Median (IQR) 62 (57–66)	Median (IQR) 8 (5–15)	184/185	46/323	138/231	Median 48	CSM
Fairey et al. [30]	2014	USA	229	1987–2008	Median (range) 65 (41–83)	NA	133/96	0/229	105/124	Median (range) 174 (2.4–253.2)	OM
Sukumar et al. [31]	2014	USA	5152	2001–2010	Mean ± SD 60 ± 7.3	Mean ± SD 6.1 ± 4.6	4341/462	3150/1653	1162/3990	Median (IQR) 26.4 (12.2–54.6)	CSS
McNeill et al. [32]	2014	Germany	575	2006–2012	Mean (range) 62 (40.3–76.5)	Mean 7.5	533/42	406/169	135/440	Median (IQR) 30 (19.3–44.0)	BRFS
Zhong et al. [33]	2012	USA	240	1993–1995	Mean 61	NA	206/34	181/59	92/148	NA	BRFS, OS
Mitchell et al. [34]	2012	USA	843	1987–1997	Median (IQR) 65 (60–69)	Median (IQR) 10.2 (4.7–23.7)	715/128	223/629	472/371	Median (range) 171.6 (0–564)	CSM, OM
Min et al. [35]	2012	Korea	830	1993–2009	Mean (range) 65.2 (41–85)	Mean (range) 12.3 (1.2–45.7)	719/109	508/322	307/523	Mean (range) 47.6 (13–87)	BRFS
Lewinshtein et al. [36]	2012	USA	91	1988–1997	mEdian (IQR) 65 (61–69)	median (IQR) 9.7 (6.1–13.4)	0/91	28/62	48/43	Median (IQR) 98.4 (54–150)	CSM
Joniau et al. [37]	2012	Germany	51	1989–2004	Mean ± SD 64.2 ± 6.4	Median (range) 16.9 (2.8–123)	NA	19/32	32/19	Median (range) 108 (11–210)	BRFS
Dorin et al. [38]	2012	USA	2487	1988–2008	NA	NA	2169/312	1783/702	658/1982	Median (range) 86.4 (12–252)	BRFS, OS
Oh et al. [39]	2011	Korea	534	2003–2008	Mean ± SD 64.9 ± 6.7	mean ± SD 11.9 ± 12.2	475/59	NA	200/334	Mean ± SD 51.2 ± 13.5	BRFS
Ku et al. [40]	2011	Korea	407	1996–2005	Mean (range) 66.5 (41.8–85.7)	Mean (range) 8.6 (0.7–142)	339/68	NA	149/258	Median (range) 18.1 (1–107.8)	BRFS
Villari et al. [41]	2010	Italy	1317	1994–2005	Median (range) 67 (38–82)	median (range) 10 (2.05–73)	NA	874/443	311/1006	Mean (range) 80.2 (4–168)	BRFS, CSS, OS
Wright et al. [42]	2010	Multi-centres	65,633	1998–2006	NA	NA	NA	56,892/8741	13,905/51,728	Median (range) 50 (1–107)	CSM

*p-PSA* preoperative prostate-specific antigen concentration, *GS* Gleason score, S*M+/SM−* surgical margin positive/surgical margin negative, *SD* standard deviation, *NA* data not applicable, *BRFS* biochemical recurrence-free survival, *CSS* cancer-specific survival, *OS* overall survival (OS), *CSM* cancer specific mortality, *OM* overall mortality

### Meta-analysis

**Fig. 2:**
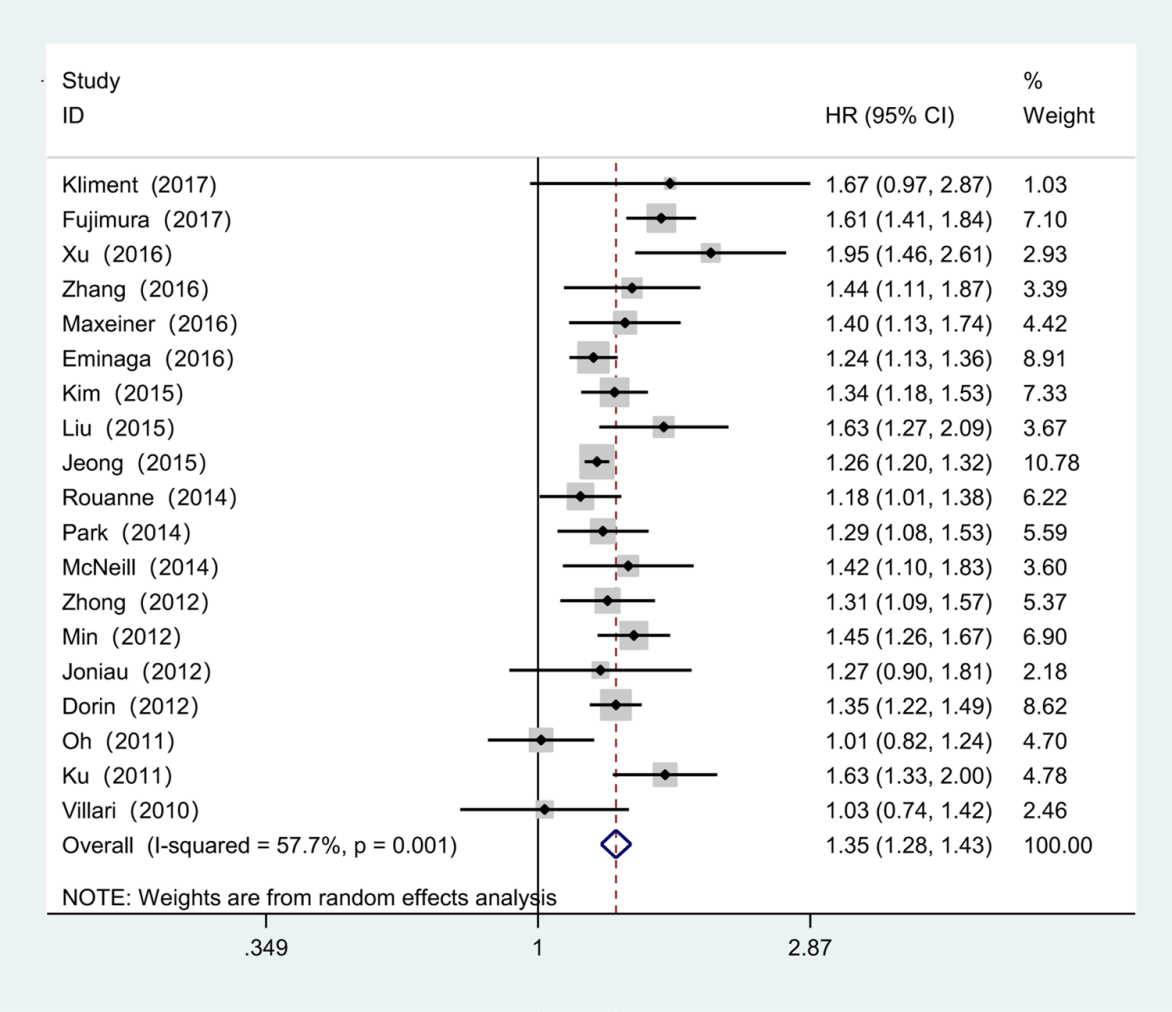
Forest plots of studies to evaluate the association between PSM and BRFS outcomes in PCa patients

**Fig. 3:**
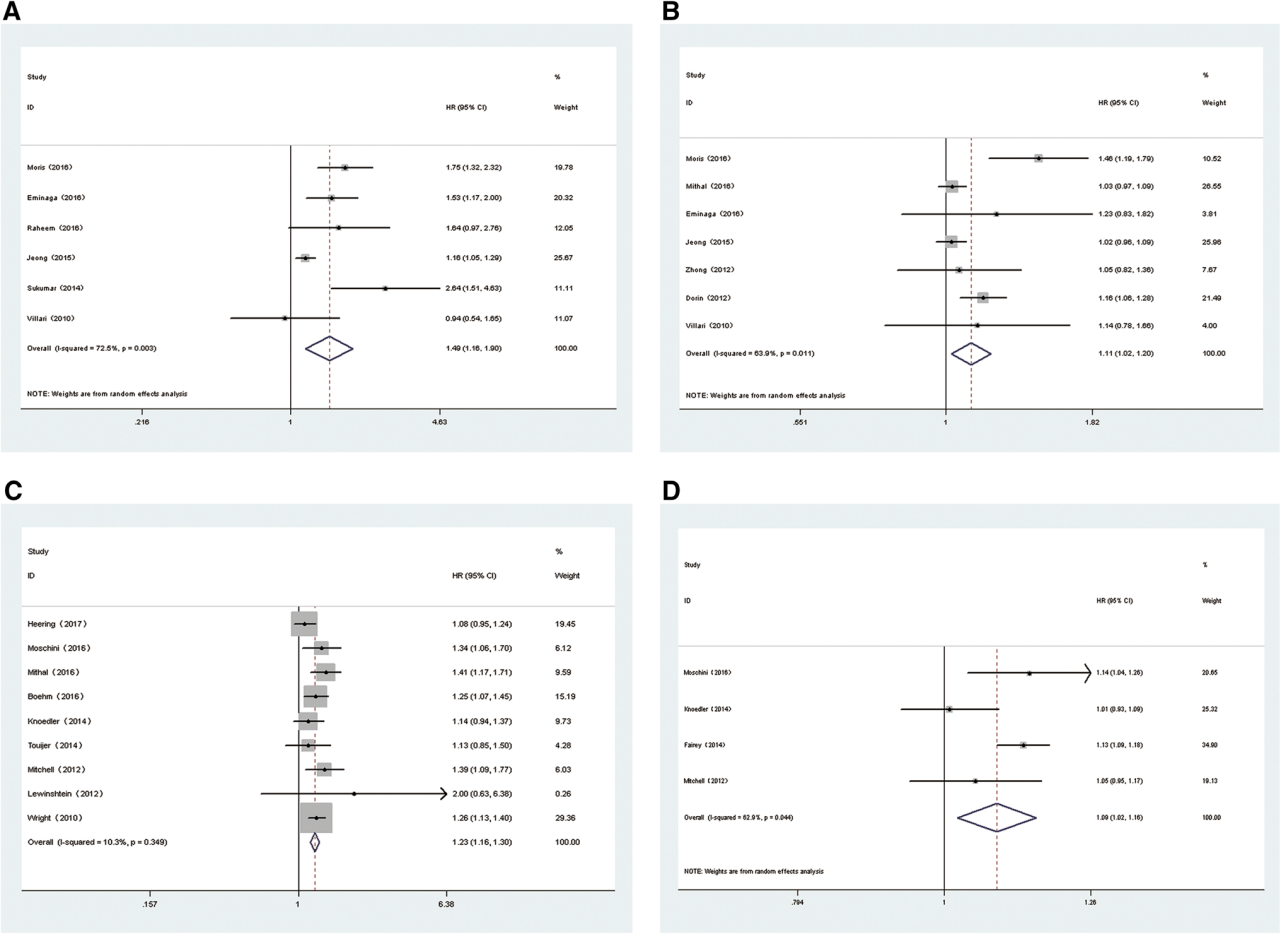
Forest plots of studies to evaluate the association between PSM and prognostic outcomes in PCa patients: **a** CSS, **b** OS, **c** CSM, **d** OM

Our meta-analysis demonstrated that a PSM in PCa was associated with poorer BRFS (RE HR = 1.35, 95% CI 1.28–1.48, *p* < 0.001, ***I***^2^ = 57.7%, *P*_heterogeneity_ = 0.001, Fig. 2), CSS (RE HR = 1.49, 95% CI 1.16–1.90, *p* = 0.001, ***I***^2^ = 72.5%, *P*_heterogeneity_ = 0.003, Fig. 3a) and OS (RE HR = 1.11, 95% CI 1.02–1.20, p = 0.014, ***I***^2^ = 63.9%, *P*_heterogeneity_ = 0.011, Fig. 3b). In addition, patients with a PSM were found to have an increased risk in terms of CSM (FE HR = 1.23, 95% CI 1.16–1.30, *p* < 0.001, ***I***^2^ = 10.3%, *P*_heterogeneity_ = 0.359, Fig. 3c) and OM (RE HR = 1.09, 95% CI 1.02–1.16, *p* = 0.009, ***I***^2^ = 62.9%, *P*_heterogeneity_ = 0.044, Fig. 3d). To explore the source of heterogeneity for BRFS, CSS, OS and OM, subgroup analyses stratified by geographical region, date of publication, mean age, sample size, mean p-PSA, median follow-up and aRT (yes/no) were performed. The results of subgroup analyses again suggested a PSM as a prognostic factor despite heterogeneity among some groups (Table 2).

**Table 2:** Summary and subgroup analysis for the eligible studies

Analysis specification	No. of studies	Study heterogeneity		Effects model	Pooled HR (95% CI)	*P* value
		*I*^2^ (%)	*P*_heterogeneit_			
BRFS						
Overall	19	57.7	0.001	Random	1.35 (1.28, 1.43)	< 0.001
Geographical region						
Asia	9	65.6	0.003	Random	1.44 (1.30, 1.61)	< 0.001
Other regions	10	0	0.634	Fixed	1.27 (1.22, 1.31)	< 0.001
Date of publication						
≥ 2015	9	68.3	0.001	Random	1.42 (1.29, 1.55)	< 0.001
< 2015	10	48	0.044	Random	1.30 (1.20, 1.41)	< 0.001
Mean age (years)						
≥ 65	9	75.7	< 0.001	Random	1.43 (1.28, 1.60)	< 0.001
< 65	7	19.4	0.282	Fixed	1.26 (1.16, 1.37)	< 0.001
Sample size (cases)						
≥ 800	7	65.9	0.007	Random	1.33 (1.23, 1.43)	< 0.001
< 800	12	53	0.016	Random	1.38 (1.26, 1.52)	< 0.001
Mean p-PSA (ng/ml)						
≥ 10	8	65.6	0.005	Random	1.32 (1.13, 1.53)	< 0.001
< 10	7	68.1	0.005	Random	1.38 (1.27, 1.50)	< 0.001
Median follow-up						
≥ 65 months	6	0	0.421	Fixed	1.27 (1.22, 1.32)	< 0.001
< 65 months	11	59.5	0.006	Random	1.39 (1.27, 1.53)	< 0.001
Adjuvant radiotherapy						
Yes	2	0	0.628	Fixed	1.37 (1.11, 1.68)	0.003
No	16	64	< 0.001	Random	1.35 (1.27, 1.44)	< 0.001
CSS						
Overall	6	72.5	0.003	Random	1.49 (1.16, 1.90)	0.001
Geographical region						
Other regions	5	76.8	0.002	Random	1.47 (1.12, 1.92)	0.005
Date of publication						
≥ 2015	4	71.5	0.015	Random	1.45 (1.14, 1.84)	0.003
< 2015	2	84.6	0.011	Random	1.58 (0.74, 4.34)	0.377
Mean age (years)						
≥ 65	2	73.1	0.054	Random	1.35 (0.74, 2.45)	0.328
< 65	4	74.9	0.007	Random	1.54 (1.13, 2.09)	0.006
Mean p-PSA (ng/ml)						
≥ 10	2	73.1	0.054	Random	1.35 (0.74, 2.45)	0.328
< 10	4	74.9	0.007	Random	1.54 (1.13, 2.09)	0.006
Median follow-up						
≥ 65 months	2	0	0.472	Fixed	1.15 (1.04, 1.28)	0.006
< 65 months	4	1.8	0.383	Fixed	1.71 (1.43, 2.04)	< 0.001
OM						
Overall	4	62.9	0.044	Random	1.09 (1.02, 1.16)	0.009
Date of publication						
< 2015	3	73.3	0.024	Random	1.07 (0.99, 1.18)	0.002
Mean age (years)						
≥ 65	2	41.6	0.191	Fixed	1.11 (1.04, 1.79)	0.004
Sample size (cases)						
≥ 800	3	49.5	0.138	Fixed	1.06 (0.99, 1.14)	0.115
Mean p-PSA (ng/ml)						
≥ 10	2	26.4	0.244	Fixed	1.10 (1.01, 1.19)	0.026
Adjuvant radiotherapy						
Yes	3	0	0.396	Fixed	1.12 (1.08, 1.16)	< 0.001
OS						
Overall	7	63.9	0.011	Random	1.11 (1.02, 1.20)	0.014
Date of publication						
≥ 2015	4	75	0.007	Random	1.10 (0.99, 1.23)	0.082
< 2015	3	0	0.772	Fixed	1.15 (1.05, 1.25)	0.002
Mean age (years)						
≥ 65	2	23.8	0.252	Fixed	1.36 (1.09, 1.70)	0.007
< 65	4	0	0.827	Fixed	1.03 (0.99, 1.07)	0.212
Sample size (cases)						
≥ 800	6	69.9	0.005	Random	1.11 (1.02, 1.22)	0.016
Mean p-PSA (ng/ml)						
< 10	3	0	0.572	Fixed	1.03 (0.97, 1.09)	0.358
Median follow-up						
≥ 65 months	4	50.4	0.109	Fixed	1.06 (0.99, 1.13)	0.074
< 65 months	2	0	0.444	Fixed	1.41 (1.18, 1.69)	< 0.001

**Fig. 4:**
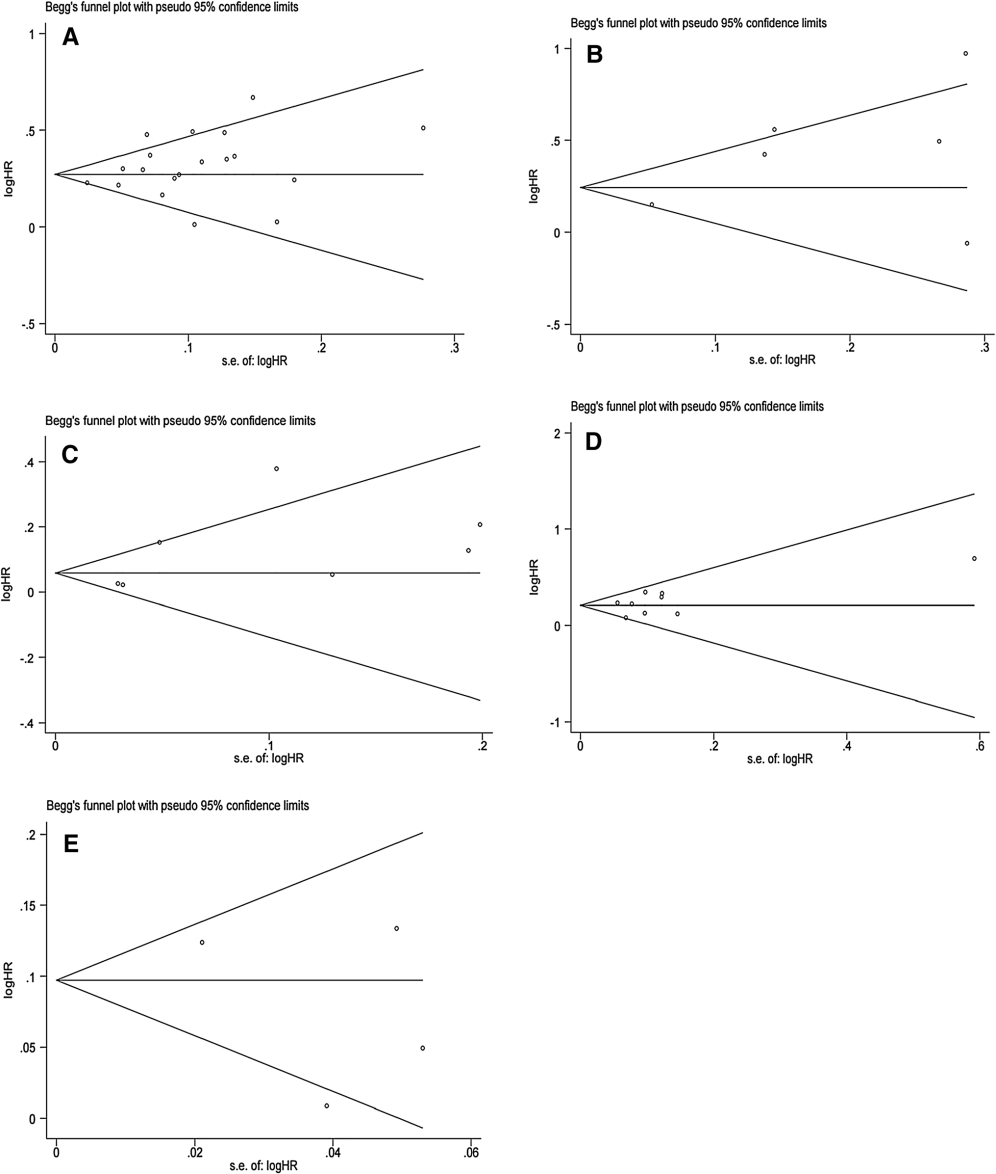
Funnel plots for evaluating publication bias of the hazard ratios (HRs): **a** BRFS, **b** CSS, **c** OS, **d** CSM, **e** OM

In sensitivity analyses, excluding one study at a time, the pooled HR for BRFS ranged from 1.33 (95% CI 1.26–1.41) to 1.37 (95% CI 1.30–1.45). Similarly, the pooled HR for CSS ranged from 1.38 (95% CI 1.11–1.72) to 1.62 (95% CI 1.28–2.05), the pooled HR for OS ranged from 1.06 (95% CI 1.00–1.11) to 1.15 (95% CI 1.03–1.29), the pooled HR for CSM ranged from 1.21 (95% CI 1.14–1.29) to 1.27 (95% CI 1.19–1.35) and the pooled HR for OM ranged from 1.06 (95% CI 0.98–1.14) to 1.12 (95% CI 1.08–1.16) (Supplementary Figure S1–5). These results indicated that the findings were reliable and robust. In addition, no statistical evidence of publication bias was found in this meta-analysis, as assessed by Egger’s tests for BRFS (*p* Egger = 0.108, Fig. 4a), CSS (*p* Egger = 0.146, Fig. 4b), OS (*p* Egger = 0.145, Fig. 4c), CSM (*p* Egger = 0.353, Fig. 4d) and OM (*p* Egger = 0.457, Fig. 4e).

## Discussion

Despite diverse multimodality treatment options and extensive studies, PCa remains a major health burden in men, and its diverse clinical outcomes regarding progression is a challenge to be addressed. As a result, various factors, including pathologic features and novel molecular biomarkers, are currently regarded as being useful for predicting the prognostic outcomes of RP. Nevertheless, PCa has been shown to be characterised by unique biological features and heterogeneous genetic backgrounds, indicating the limitations for predicting postoperative prognostic outcomes in patients with localised PCa [43].

RP with pelvic lymph node dissection is the standard of care for localised PCa, with the goals of providing good oncologic and functional outcomes, especially in patients with good life expectancy. However, a proportion of patients inevitably demonstrate adverse pathologic features such as PSMs, seminal vesicle invasion [44], lymph node metastasis [45] and perineural invasion [46]. The reported incidence of PSMs, notwithstanding its significant decrease with RP because of the advances in surgical techniques, signifies locally adverse pathology, and PSMs remain an ominous prognostic factor [47, 48]; moreover, the management of patients with PSMs remains challenging. Furthermore, the impact of PSMs on control of PCa has been controversial. For example, in an analysis of the pathological reports of 65,633 specimens from RPs, Wright et al. demonstrated the independent role of a PSM in PCa [26]. Subsequently, Alkhateeb et al. [49] reported that a PSM was an independent predictor of BRFS in patients with intermediate- and high-risk PCa. However, Mithal et al. [8] reported that a PSM was significantly associated with all adverse outcomes in unadjusted models, although PSMs were only associated with increased risk of BCR (HR = 1.98, *p *< 0.001) and not with castration-resistant disease, metastases, or CSM (HR ≤ 1.29, *p* > 0.18) after adjusting for demographic and pathological characteristics.

Patients with BCR following RP have been shown to be at increased risk for subsequent metastases and death. However, BCR represents an early event in the natural history of PCa with heterogeneous outcomes, and BCR does not systematically translate into clinical progression [42]. Although previous studies have found that PSMs are associated with an increased risk of BCR, their association with more clinically robust endpoints is still controversial [50]. The prognostic heterogeneity may often have been incompletely characterised due to limitations in sample size, and only a large study with enough events can evaluate whether a PSM is an independent predictor of clinical outcome. In this meta-analysis, we synthesised 32 studies with a large sample of 75,589 patients, including 31,421 PSM patients (22.2%), to explore the relationship between PSMs and oncologic outcomes in localised PCa.

To the best of our knowledge, the present study was the first to systematically evaluate the prognostic value of PSM in patients with PCa, and the data showed that a PSM was a predictor for BRFS (HR = 1.35, *p* < 0.001), CSS (HR = 1.49, *p* = 0.001), OS (HR = 1.11, *p* = 0.014), CSM (HR = 1.23, *p* < 0.001) and OM (HR = 1.09, *p* = 0.009). The findings were consistently independent of geographical region, publication year, age, sample size, p-PSA, follow-up duration and aRT (yes/no). Sensitivity analyses indicated that the findings were reliable and robust. In addition, there was no evidence of significant publication bias in these analyses according to Begg’s tests. Although there was no evidence of heterogeneity in terms of CSM, significant heterogeneity was detected in the analysis of the BRFS, CSS, OS and OM models. To further explore the source of heterogeneity, subgroup analyses were conducted. Our data showed that the significant variations were reduced within some items.

Although we used a systematic method to perform the present study, the following limitations also should be taken into account. First, the applied methods for detecting PSMs in the pathologic specimen were varied in the included studies, which may cause heterogeneity among the studies. Second, substantial heterogeneity was observed in the meta-analysis; although we chose the RE model according to heterogeneity, it still existed in our studies. The heterogeneity was probably caused by differences in factors such as the patients’ characteristics and different durations of follow-up. Third, we only included published studies written in English, and grey literature was not included, which may cause selection bias. Fourth, all the included studies were retrospective cohort studies, and data extracted from those studies may have led to inherent potential bias.

Nevertheless, the present study has several key strengths. First, the meta-analysis included 32 studies with a large sample size to detect more stable associations between PSMs and clinical outcomes of PCa patients. Second, with the strict inclusion and exclusion criteria, we extracted available data from relevant studies. Furthermore, the results were found to be reliable and robust through subgroup and sensitivity analyses. Therefore, PSM determination, with excellent accessibility and low costs, warrants wider application in patients with PCa for risk stratification and decision-making of individualised treatment.

In conclusion, the results of this meta-analysis demonstrated that the finding of PSMs by histopathology is closely associated with poor survival in patients with PCa. Due to limitations in this study, large-scale, multicentre prospective studies with standardised methods and long-term follow-up are needed to verify our results.

### Availability of materials and data

All data generated or analysed during this study are included in this published article (and its supplementary information files).

## Electronic supplementary material

Below is the link to the electronic supplementary material.

Supplementary material 1 (DOCX 15 kb)

Supplementary material 2 (TIFF 6285 kb)
