# Supplementary material for: Surgical margin status and its impact on prostate cancer prognosis after radical prostatectomy: a meta-analysis
Source: World J Urol. 2018 May 15;36(11):1803–15. doi: 10.1007/s00345-018-2333-4 (PMC6208659; doi:10.1007/s00345-018-2333-4)
Supplement: Supplementary file 1 — Supplementary material 1 (DOCX 15 kb) [file 345_2018_2333_MOESM1_ESM.docx]

**Supplementary Table S1. Quality assessment of cohort studies included in this meta- analysis**

| **Study** | **Representativeness of the exposed cohort** | **Selection of the unexposed cohort** | **Ascertainment of exposure** | **Outcome of interest not present at start of study** | **Control for important factor or additional factor** | **Outcome assessment** | **Follow-up long enough for outcomes to occur** | **Adequacy of follow-up of cohort** | **Total quality scores** |
| --- | --- | --- | --- | --- | --- | --- | --- | --- | --- |
| Kliment et al | ★ | ★ | ★ | ★ | ★ | ★ | ★ | ★ | 8 |
| Fujimura et al | ★ | ★ | ★ | ★ | ★★ | ★ | — | ★ | 8 |
| Heering et al | ★ | ★ | ★ | ★ | ★★ | ★ | ★ | ★ | 9 |
| Zhang et al | ★ | ★ | ★ | ★ | ★ | ★ | ★ | ★ | 8 |
| Xu et al | ★ | — | ★ | ★ | ★ | ★ | ★ | ★ | 7 |
| Moschini et al | ★ | ★ | ★ | ★ | ★★ | ★ | ★ | ★ | 9 |
| Raheem et al | ★ | ★ | ★ | ★ | ★★ | ★ | ★ | ★ | 9 |
| Moris et al | ★ | ★ | ★ | ★ | ★★ | ★ | ★ | ★ | 9 |
| Boehm et al | ★ | ★ | ★ | ★ | ★ | ★ | ★ | ★ | 8 |
| Mithal et al | ★ | ★ | ★ | ★ | ★ | ★ | ★ | ★ | 8 |
| Maxeiner et al | ★ | ★ | ★ | ★ | ★★ | ★ | ★ | ★ | 9 |
| Eminaga et al | ★ | ★ | ★ | ★ | ★★ | ★ | ★ | ★ | 9 |
| Liu et al | ★ | ★ | ★ | ★ | ★ | ★ | ★ | ★ | 8 |
| Kim et al | ★ | ★ | ★ | ★ | ★ | ★ | ★ | ★ | 8 |
| Jeong et al | ★ | ★ | — | ★ | ★ | ★ | ★ | ★ | 7 |
| Rouanne et al | ★ | ★ | ★ | ★ | ★ | ★ | ★ | ★ | 8 |
| Park et al | ★ | ★ | ★ | ★ | ★★ | ★ | ★ | ★ | 9 |
| Knoedler et al | ★ | ★ | ★ | ★ | ★★ | ★ | ★ | ★ | 9 |
| Touijer et al | ★ | ★ | ★ | ★ | ★ | ★ | ★ | ★ | 8 |
| Fairey et al | ★ | ★ | ★ | ★ | ★ | ★ | ★ | ★ | 8 |
| Sukumar et al | ★ | ★ | ★ | ★ | ★★ | ★ | ★ | ★ | 9 |
| McNeill et al | ★ | ★ | ★ | ★ | ★ | ★ | ★ | ★ | 8 |
| Zhong et al | ★ | ★ | ★ | ★ | ★ | ★ | — | ★ | 7 |
| Mitchell et al | ★ | ★ | ★ | ★ | ★ | ★ | ★ | ★ | 8 |
| Min et al | ★ | ★ | ★ | ★ | ★ | ★ | ★ | ★ | 8 |
| Lewinshtein et al | ★ | ★ | ★ | ★ | ★★ | ★ | ★ | ★ | 9 |
| Joniau et al | ★ | ★ | ★ | ★ | ★★ | ★ | ★ | ★ | 9 |
| Dorin et al | ★ | ★ | — | ★ | ★★ | ★ | ★ | ★ | 9 |
| Oh et al | ★ | ★ | ★ | ★ | ★★ | ★ | ★ | ★ | 9 |
| Ku et al | ★ | ★ | ★ | ★ | ★★ | ★ | ★ | ★ | 9 |
| Villari et al | ★ | ★ | ★ | ★ | ★ | ★ | ★ | ★ | 8 |
| Wright et al | ★ | ★ | ★ | ★ | ★ | ★ | ★ | ★ | 8 |
