# Supplementary figures and images for: Surgical margin status and its impact on prostate cancer prognosis after radical prostatectomy: a meta-analysis
Source: World J Urol. 2018 May 15;36(11):1803–15. doi: 10.1007/s00345-018-2333-4 (PMC6208659; doi:10.1007/s00345-018-2333-4)

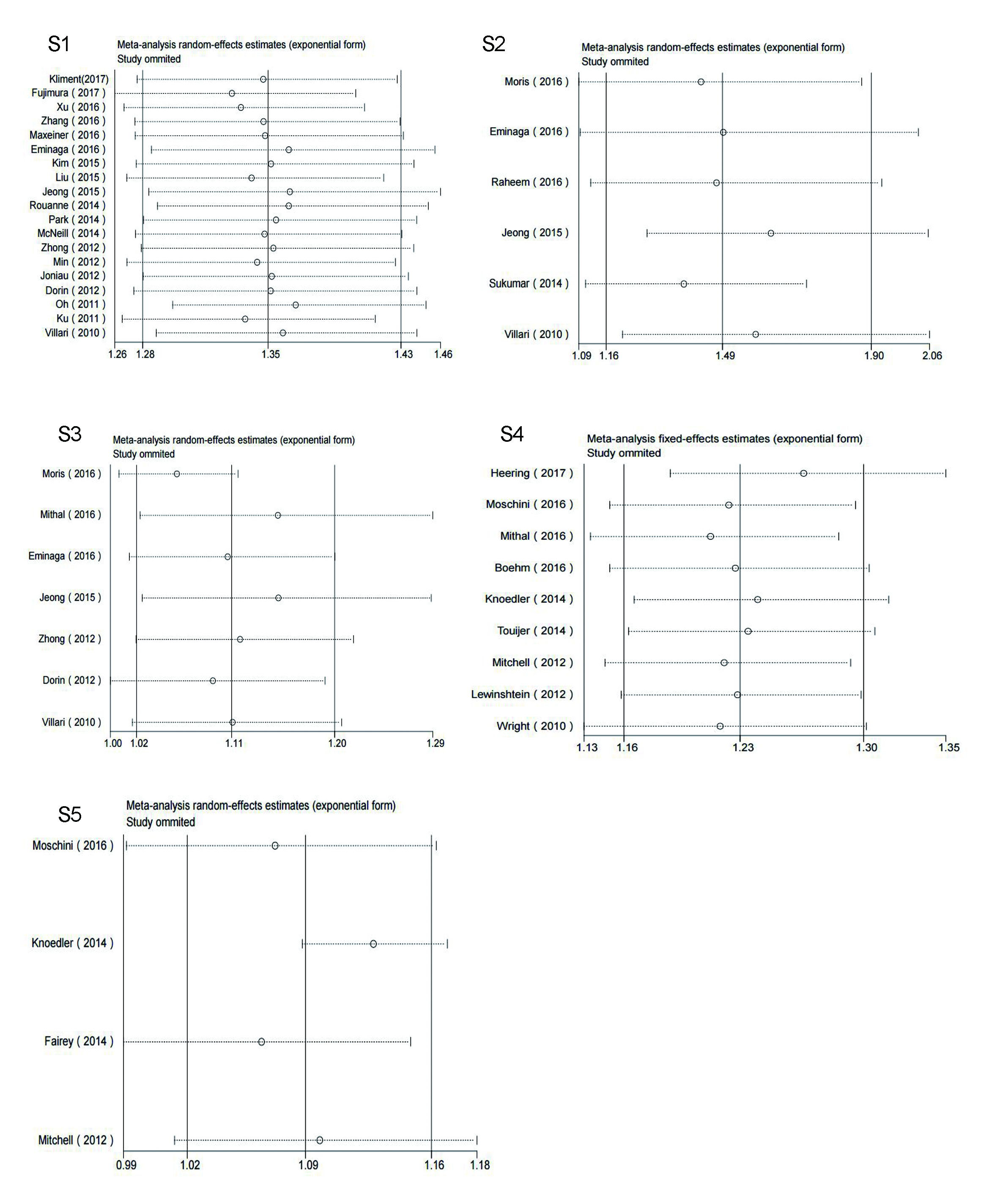

Supplement: Supplementary file 2 — Supplementary material 2 (TIFF 6285 kb) [file 345_2018_2333_MOESM2_ESM.tif]
